# 

**Published:** 1906-12

**Authors:** 


### CONTENTS.

| | Page |
|---|---|
| X-Ray Therapeutics. By JAMES TAYLOR, M.R.C.S. ... ... ... ... | 289 |
| Fibroma of the Abdominal Wall. By J. LACY FIRTH, M.S. Lond., F.R.C.S. ... ... ... ... ... ... ... ... ... | 304 |
| The Preservation of the Limb in the Treatment of Sarcoma of Bone. By CHARLES A. MORTON, F.R.C.S ... ... ... ... | 308 |
| Proteins and Protein Metabolism. By J. M. FORTESCUE-BRICKDALE, M.A., M.D. Oxon. ... ... ... ... ... ... ... ... | 318 |
| The Long Fox Lecture: Dermatitis from Without and Dermatitis from Within. By ALFRED HARRISON, M.B. Lond. ... ... ... | 325 |
| Progress of the Medical Sciences: | |
| Medicine. CAREY COOMBS, M.D. Lond. ... ... ... ... ... | 344 |
| Surgery. HAROLD F. MOLE, F.R.C.S.... ... ... ... ... | 348 |
| Dermatology. J A. NIXON, M.B. Cantab., M.R.C.P. ... ... | 353 |
| Reviews of Books ... ... ... ... ... ... ... ... ... | 359 |
| Editorial Notes ... ... ... ... ... ... ... ... ... | 370 |
| Notes on Preparations for the Sick ... ... ... ... ... | 376 |
| The Library of the Bristol Medico-Chirurgical Society ... ... | 379 |
| Meetings of Societies ... ... ... ... ... ... ... ... | 382 |
| Obituary Notices ... ... ... ... ... ... ... ... ... | 386 |
| Local Medical Notes ... ... ... ... ... ... ... ... | 391 |

